# Circulating microRNA’s as a diagnostic tool for hepatocellular carcinoma in a hyper endemic HIV setting, KwaZulu-Natal, South Africa: a case control study protocol focusing on viral etiology

**DOI:** 10.1186/s12885-017-3915-z

**Published:** 2017-12-28

**Authors:** K. Sartorius, B. Sartorius, A. Kramvis, E. Singh, A. Turchinovich, B. Burwinkel, T. Madiba, C. A. Winkler

**Affiliations:** 1 Department of Public Health Medicine, School of Nursing and Public Health, University of KwaZulu-Natal, Durban, 4041 South Africa; 2 Faculty of Commerce, Law and Management, University of the Witwatersrand, Johannesburg, South Africa; 3 UKZN Gastrointestinal Cancer Research Centre (GICRC), Durban, South Africa; 4 Hepatitis Virus Diversity Research Unit, Department of Internal Medicine, School of Clinical Medicine, Faculty of Health Sciences, University of the Witwatersrand, Parktown, Johannesburg, South Africa; 5 South African National Cancer Registry, National Health Laboratory Service, Johannesburg, South Africa; 6 Molecular Epidemiology Group, German Cancer Research Centre, Heidelberg, Germany; 7 SciBerg e.Kfm, Mannheim, Germany; 8 Basic Research Laboratory, Centre for Cancer Research, National Cancer Institute, Leidos Biomedical Research, Inc. Frederick Nat. Lab. for Cancer Research, Frederick, MD USA

**Keywords:** Hepatocellular carcinoma, miRNA, Biomarker, Diagnosis, Staging, HBV, HIV

## Abstract

**Background:**

A wide range of studies has investigated the diagnostic proficiency of extracellular microRNAs (miRNAs) in hepatocellular cancer (HCC). HCC is expected to increase in Sub-Saharan Africa (SSA), due to endemic levels of viral infection (HBV/HIV), ageing and changing lifestyles. This unique aetiological background provides an opportunity for investigating potentially novel circulating miRNAs as biomarkers for HCC in a prospective study in South Africa.

**Methods:**

This study will recruit HCC patients from two South African cancer hospitals, situated in Durban and Pietermaritzburg in the province of KwaZulu-Natal. These cases will include both HBV mono-infected and HBV/HIV co-infected HCC cases. The control group will consist of two (2) age and sex-matched healthy population controls per HCC case randomly selected from a Durban based laboratory. The controls will exclude patients if they have any evidence of chronic liver disease. A standardised reporting approach will be adopted to detect, quantify and normalize the level of circulating miRNAs in the blood sera of HCC cases and their controls. Reverse transcription quantitative polymerase chain reaction (RT-qPCR) will be employed to quantity extracellular miRNAs. Differences in concentration of relevant miRNA by case/control status will be assessed using the Wilcoxon rank-sum (Mann-Whitney U) test. Adjustment for multiple testing (Bonferroni correction), receiver operating curves (ROC) and optimal breakpoint analyses will be employed to identify potential thresholds for the differentiation of miRNA levels of HCC cases and their controls.

**Discussion:**

Although there is a growing base of literature regarding the role of circulating miRNAs as biomarkers, this promising field remains a ‘work in progress’. The aetiology of HBV infection in HCC is well understood, as well as it’s role in miRNA deregulation, however, the mediating role of HIV infection is unknown. HCC incidence in SSA, including South Africa, is expected to increase significantly in the next decade. A combination of factors, therefore, offers a unique opportunity to identify candidate circulating miRNAs as potential biomarkers for HBV/HIV infected HCC.

**Electronic supplementary material:**

The online version of this article (10.1186/s12885-017-3915-z) contains supplementary material, which is available to authorized users.

## Background

MicroRNAs (miRNAs) are (mostly) endogenously developed fragments of single stranded non-coding RNA (19-25 nucleotides) that regulate more than 50% of all cell specific protein translation. The deregulation of miRNAs is linked to cancer because they play a role in modulating target genes responsible for cell proliferation, apoptosis, DNA repair, invasion and metastasis [1]. The sensitivity of miRNA expression (transcription) alteration in cancer incidence is underlined by the location of their parent genes, often found in fragile chromosomal regions that exhibit DNA amplification, deletions and translocations which deregulate miRNA expression [2]. Circulating miRNAs have been proposed as promising biomarkers for cancer pathologies because of their abundance in sera, as well as their stability under extreme conditions [3, 4]. Serum miRNAs are resistant to ribonuclease digestion because they are protected in protein complexes or in membranous micro-vesicles that transport them in the circulatory system [1, 5]. The stability of cell free, circulating miRNAs is underlined by the fact that successful quantification has been observed in samples stored up to 10 years at −80 degrees C [1, 6]. Despite considerable investigation of extracellular miRNAs, the use of miRNAs as biomarkers of cancer is still regarded as a ‘work in progress’ and mostly restricted to research programs [5, 7]. Continuing technological developments, however, like 2nd generation sequencing, as well as a better understanding of the pathobiological role of miRNAs, underline their future promise as clinical biomarkers [7, 8].

A wide range of studies has investigated the diagnostic proficiency of circulating miRNAs in liver diseases, including hepatocellular carcinoma (HCC), chronic hepatitis (CH), non-alcoholic fatty liver disease (NAFLD), liver toxicity, cirrhosis and non-alcoholic steatohepatitis [9–12]. The strong causal association between HCC and CH continues to influence HCC incidence [3, 13] while emerging studies explain the biological role of viral miRNAs [14]. Sub-Saharan Africa, and South Africa in particular, is an endemic region for both HBV and HIV infection, as well as rapid urbanization and lifestyle changes [15]. The aim of this study is to investigate circulating miRNAs as biomarkers for HCC in South Africa against a background of both HIV/HBV mono and co-infection.

### Empirical evidence of circulating miRNAs in HCC

In hepatocellular carcinoma (HCC), a range of miRNAs is deregulated in response to cancer cells that promote aberrant expressions in their target genes [16]. HCC deregulates the expression of circulating miRNAs (upwards or downwards) to inversely influence the expression of target mRNAs/ specific genes involved in cell cycle regulation, apoptosis, DNA repair, invasion and metastasis [2]. In HCC development, the miRNA mediated expression of mRNA can have either oncogenic effects or promote a loss of tumor suppressor function [2, 14, 16].

Emerging evidence indicates multiple miRNAs are deregulated in HCC. A recent reviewed collated a wide range of studies to collectively indicate 55 miRNAs that are down-regulated and 41 miRNAs that are up-regulated in HCC [16]. The presence and proficiency of circulating miRNA as biomarkers for HCC, have been tested both individually, as well as in selected groups. Examples of deregulated circulating miRNAs, identified in numerous studies, include miR-10a, miR-21, miR-23a/b, miR-25, miR-26a/b, miR-122, miR-125b, miR-192, miR-222, miR-223, miR-342-3p, miR-375, miR-423, miR-801, miR-885-5p, and miR-Let-7f [3]. It has been suggested that miR-122a is the most abundant miRNA in hepatocytes [9], that it is a reliable marker of viral infection [17] and it is down-regulated in ~70% of HCC [18]. MiR-500 is also abundantly expressed in liver cancer cell lines and deregulation of miRNA occurs in ~45% of HCC cases [19].

### HCC, viral infection and circulating miRNAs

Viruses encode their own sets of miRNA which are used to control the expression of their host’s genes [20]. The ability of a virus to package its own miRNAs into exosomes and transport them to non-infected cells was first demonstrated by the EBV virus [21]. Both viral transcripts and proteins can affect host miRNA expression, which can modulate viral and/or host protein expression [22]. MiRNAs can bind to viral genomes or transcripts and regulate viral infection and, conversely, viral infection (e.g. HIV/HCV) can modulate host-cell microRNA machinery [23]. The role of miRNAs in viral infection is being demonstrated in an increasing number of studies. MiR-122, for instance, down-regulates HBV replication by binding to the viral target sequence [24] and, conversely, binds to the HCV genome to increase viral translation and replication [25–27]. MiR-199a and miR-210 bind to different sites on mRNA coding of HBsAg, reducing HBsAg expression in HepG2 2.2.15 cells [28]. MiR-15b has also been shown to modulate HBV replication by targeting the hepatocyte nuclear factor 1α (HNF1α) [29], while miR-130a expression is increased in HCV infection.

Two review papers, summarizing a wide range of studies [2, 3], identified a marker group of seven circulating miRNAs, including miR-122, miR-192, miR-223, miR-21, miR-26a, miR-27a, miR-801 that were able to distinguish between HCC, HBV, cirrhosis and healthy controls, as well as identify HCC tumor stages. Others have shown that serum levels of miR-10a and miR-125b were lower in HBV infected HCC patients than in chronic hepatitis B (CHB) patients and that a triplet of circulating miRNAs [namely miR-375, miR-25, miR-Let-7f] were able to diagnose HCC with ~98% accuracy [3]. Circulating miR-21 was also higher in HCC than chronic hepatitis patients and healthy controls; furthermore, its levels correlated with miR-21 expressed in HCC tumor tissue and it had better diagnostic sensitivity than alpha fetoprotein (AFP) [2, 3]. In another study, it was found that serum miR-21, −122, and −223 were higher in HCC and CH versus controls, whereas miR-122 and miR-21 were higher for CH than HCC but not miR-223 [30].

### Biological relevance of deregulated miRNAs in HCC

Various studies are increasingly beginning to explain the biology of specific circulating miRNAs and their potential role in HCC, with respect to cell proliferation, angiogenesis and metastasis (see Additional file 1: Table S1). Cell proliferation in HCC is promoted by the downregulation of miR-26a, which acts as a partner with miR-195 to overcome the G1/S cell cycle blockade through the repression of E2F expression. Cell proliferation in HCC is also influenced by upregulated miRNAs (e.g. miR-21, miR-216a) that promote tyrosine kinase by downregulating the PTEN tumour suppressor protein [14]. MiR-122, for example, can inhibit angiogenesis and intrahepatic metastasis by suppressing the expression of the tumour necrosis factor-α- converting enzyme (TACE) [14]. The metastasis of HCC is also influenced by miR-10a which regulates ephrin-type-A-receptor-4 mediated mesenchymal transition [14, 16].

### Controversial issues in circulating miRNA research

Recent research indicates that miRNAs are found in all cellular components, where they regulate transcription, translation, alternative splicing and DNA repair [31]. A number of unsolved issues continues to delay the use of circulating miRNAs as viable cancer/ disease biomarkers. The mechanism of their generation and possible pathways is still being investigated [31] and their biological role as messenger miRNAs in signaling, remains unclear [32, 33]. A question also remains as to whether only certain types of extracellular vesicles (e.g. exosomes) transport messenger MiRNAs and others merely transport small RNA as debris [7, 34]. In addition, the elevation of extracellular miRNAs in the blood sera of cancer patients has been attributed to general conditions like inflammation, rather than as a result of an early stage tumor [7] Another issue is that recent reviews suggest that methodological problems in many earlier studies re non standardization of sample collection, sample quality control, RNA isolation, RT-qPCR and data normalization, have rendered their findings questionable [7, 17, 35]. Finally, the ability of miRNAs to silence their target mRNAs, is also influenced by polymorphisms in their parent genes that cause small changes in the miRNA nucleotides, thus inducing a change in their ability to bind to mRNA targets [16].

## Methods

Non-standard data collection and analysis have precluded the publication of many miRNA studies in high impact journals. This study will adopt the suggested checklist proposed by Kirschner et al., that covers sample collection, sample quality control, RNA isolation, RT-qPCR and data normalization [35].

### Study population, data/sera and sample size

Data for the study are prospective and will be recruited from all HCC patients reporting to two South African hospitals, namely, Inkosi Albert Luthuli Central Hospital (IALCH) in Durban, KwaZulu-Natal and Greys hospital in Pietermaritzburg, KwaZulu-Natal. Prospective HCC cases will be collected from January 2017 until 2020. Each patient will complete a questionnaire that provides both demographic and lifestyle data. Each patient will be tested for HIV/HBV infection, as well as routine markers assessed for each HCC patient, including AFP and ALT/AST. A total of 200 HCC cases is estimated in the collection period and 400 healthy controls which are age and sex matched.

### Blood collection for miRNA assessment

Blood will be collected from consenting patients that are diagnosed with HCC in the oncology departments of the IALCH and Greys hospital. An 18-20 gauge syringe needle will be used to obtain the blood sample, which will be deposited in miRNeasy collection tubes (1.5 ml to 2 ml). Within 60 min of blood collection samples being taken, they will be centrifuged at 2500 g for 20 min at room temperature. Plasma supernatant will be removed and samples frozen as 500 ul aliquots and stored at −80 deg. C in pre-determined pools that relate to HCC stage 1-1 V.

### Assessment of haemolysis

The level of haemolysis will be assessed by using a spectrophotometer and samples will be classified as being haemolysed if the level of free haemoglobin (OD_414_) exceeds a cut off (0.2). This is important in HCC with respect to miRs 15a/−16/−210 [16], which are altered by the haemolysis of red blood cells.

### RNA isolation

The study will use a Trizol + miRNeasy modified method to isolate miRNA from serum. Critique of different approaches indicates that non-column based purification using reagents like TRIzol or QIAzol, might be more efficient with respect to samples with a low RNA concentration [35]. The miRNeasy kit, however, is superior in isolating small endogenous RNA that is moderately to abundantly expressed, as well as superior with respect to isolating exogenous spike-ins (*C. elegans*) [35]. The isolation of RNA will commence by defrosting the serum sample on ice (only invert to mix and no vortex) and transferring 400ul of serum into a fresh tube (2 ml). 1200ul of Tri-Reagent LS (Invitrogen or Sigma) will be added and the sample vortexed, and then incubated for 5 min at room temperature (RT), before adding 2 ul cel-mir-39 (5fmol/ul) and 10μg glycogen (20 mg/ml, RNA grade). The sample will be vortexed again before adding 320ul chloroform and vortexed for 5-10 s. All samples will then be vortexed (vigorously) for 45 s and incubated at RT for 10 min. Samples will then be centrifuged at 16000 g at room temperature before carefully transferring the supernatant (≈800ul) into a fresh tube (2 ml). 1.5 volumes ethanol and mix will be added by pipetting up and down several times.

Each sample in 700ul aliquots will be added to a mini spin column (Qiagen miRNeasy Kit) and centrifuged at 16000 g for 10 s and the flow through discarded. The column will then be washed once with 700ul RWT Buffer, followed by a wash with 500ul RPE Buffer, and centrifuged at 16000 g for 15 s. The column will be washed again by 500ul RPE Buffer and further centrifuged at 16000 g for 15 s. The column will then dry spin for 1 min at maximum speed and be transferred to a fresh collection tube. Finally, 50ul of RNase free water will be added to the column, incubated for 2 min and centrifuged at 16000 for 1 min.

### RNA quantification

It is presumed that the larger sample input of 400ul will promote the detection of RNA concentration using standard spectrophotometry [35]. The RNA concentration will be measured using a Qubit RNA HS Assay kit in preference to the NanoDrop, because it is presumed that the elutes will contain contaminating proteins and polysaccharides. The Qubit kit is also specific to ssRNA.

### Reverse transcription real time quantitative PCR

This process commences with the reverse transcription (RT) of total mature miRNA (10 ng) from the pooled serum in order to synthesize cDNA using a TaqMan® microRNA Reverse Transcription Kit (catalogue number 4366596; Applied Biosystems) and Megaplex RT miRNA specific primers (catalogue number 4399966 from Applied Biosystems). The manufacturer’s protocol will be adhered to with respect to the reverse transcription of up to approximately 380 miRNAs thus ensuring the appropriate miRNA cDNA library is developed. RT will be performed using a thermo-cycler (Mastercycler Epgradient thermocycler; Eppendorf). The following specific cycling conditions will be used; 40 cycles of 16 °C for 2 min, followed by 42 °C for 1 min and then 50 °C for 1 s. In order to de-activate the transcriptase, a final cycle at 80 °C for 5 min is completed.

In order to ensure sufficient miRNA cDNA material is available for RT- PCR, cDNA libraries generated from the previous step, will be pre-amplified under supplier directions using a primer (Megaplex PreAmp primer, catalogue number 4399233; Applied Biosystems) and a PreAmp Master Mix (catalogue number 4384266; Applied Biosystems). The PreAmp primer pool selected will be based on a library of (forward) primers that have been identified for human miRNAs that mediate hepatocellular carcinoma and related cirrhotic conditions including viral infection (HBV). A universal reverse primer will be employed. The pre-amplification cycling conditions will be run under prescribed temperature and time cycles. These include a cycle at 95 °C for 10 min, a cycle of 55 °C for 2 min and a cycle of 72 °C for 2 min. This will then be followed by 12 cycles of 95 °C for 30 s and 60 °C for 4 min. Finally, the samples will then be held at 99.9 °C for 10 min.

The expression levels of miRNA are then determined by TaqMan Low Density Arrays (TLDA). The TLDA step follows the pre-amplification of the cDNA libraries [36]. TLDA commences with the dilution of product of the previous step in RNase-free water that is combined with a gene expression master mix (TaqMan). The diluted product is transferred onto a 384-well TaqMan Low Density (TLDA) microarray plate (TaqMan Human MicroRNA Array A, catalogue number 4398965; Applied Biosystems). The microarray plate incorporates a real-time TaqMan™ Array Microfluidic Card that has been customized to include up to 384 microarray ‘hits’ with primers and probes situated in each well for up to 384 miRNAs.

The supplier instruction pack will be followed for RT- qPCR using a sequence detection system (ABI PRISM 7900HT-Applied Biosystems) under a specific set of cycling conditions. These conditions in sequence are 50 °C for 2 min and 94.5 °C for 10 min. The final sequences are 40 cycles of 95 °C for 30 s and 59.7 °C for 1 min. The cycle threshold, namely, the fractional cycle number at which the fluorescence passes the fixed threshold of 0.2, will be generated by software (SDS 2.3 -Applied Biosystems). An endogenous control, Mamm *U6* is embedded in the microarray (TaqMan Human MicroRNA Arrays).

### Normalization of RT-qPCR data (Cq values)

The normalization of miRNA levels will be assessed using the average recovery of the spike-ins and this will be compared to the standard deviation of ubiquitous hepatocyte miRNAs like miR-122. The relative expression levels of miRNAs will be calculated using the comparative ΔΔ*C*_t_ method [37, 38]. The fold changes in miRNAs will be calculated by the eq. 2^−ΔΔ*C*^_*t*_. Cluster 3.0 software will be used to perform unsupervised hierarchical clustering, using Pearson’s correlation metrics and average linkage methods. Java Treeview 1.1.3 will be used to visualize the clustering results.

### Statistical analysis

All statistical calculations were performed using Stata 13.0 and/or R. The Wilcoxon rank-sum (Mann–Whitney U) test or Kruskal–Wallis test will be used to assess differences in serum concentration levels of miRNA levels by group. Receiver operating characteristic (ROC) curves and will be constructed and the area under the ROC curve (AUC) calculated, along with various performance statistics (sensitivity, specificity, PPV, NPV) based on the estimated optimal breakpoint for a given miRNA that best differentiates HCC cases from controls. Parametric Linear regression analysis may be used to examine correlations between the levels of the miRNAs and a range of HCC related variables and other liver function parameters. Non-parametric equivalents will also be employed if the assumption of the parametric linear regression approach is not upheld. Interaction terms for co-infection of HIV/HBV will be included to assess differences in miRNA profile and concentration between individuals with no infection versus discrete (singular infection) versus co-infection. A correction for multiple testing (Bonferroni correction) will be employed. An adjusted *P* value of <0.05 will be considered statistically significant.

## Discussion

Although there is a growing base of literature regarding circulating miRNA as potential biomarkers for cancer, this field can still be classified as ‘a work in progress’, largely because of the non- standardised protocols used in many earlier studies, as well as unproved hypotheses relating to the biology and role of circulating miRNAs [39, 40]. This could be especially relevant in developing settings with a high burden of oncogenic viruses (HBV/ HIV) that mediate miRNA deregulation in carcinogenesis. Given the inherent regulatory function of miRNAs, it is highly likely that many miRNAs (both host and viral), expressed in relevant tissue, influence both the biological behaviour and clinical phenotype of the tumour. The development of powerful tools for miRNA characterization and quantitation, such as qRT-PCR and Deep Sequencing, suggests that the discovery pipeline for miRNA biomarkers could potentially be more efficient than “traditional” proteomic biomarker pipelines [39]. The latter is limited by delays or bottlenecks at the antibody generation phase as well as “quantitative assay development for validation of biomarker candidates” [39].

The results from this proposed study will help identify circulating miRNAs as HCC cancer biomarkers in a setting with high HIV/HBV co-infection. As the functional roles of miRNAs in HCC development are further elucidated and better understood, we foresee that HCC specific blood-based miRNA biomarkers will be useful in both diagnosing HCC at an earlier stage, as well as assisting with predicting the clinical course and/or therapeutic response to currently available therapies.

## Additional file

Additional file 1: Table S1. MiRNA deregulation, targets and effect in HCC. (DOCX 12 kb)

## Abbreviations

BREC: Biomedical Research Ethics Committee
CH: Chronic hepatitis
HBV: Hepatitis B virus
HCC: Hepatocellular cancer
HCV: Hepatitis C virus
HIV: Human Immunodeficiency virus
IALCH: Inkosi Albert Luthuli Central Hospital
KZN: Kwazulu-Natal
NAFLD: Non-alcoholic fatty liver disease
NASH: Non-alcoholic steatohepatitis
NPV: Negative predictive value
PLC: Primary liver cancer
PPV: Positive predictive value
ROC: Receiver operating characteristic
RT-PCR: Reverse transcription polymerase chain reaction
SSA: Sub-Saharan Africa
UTR: Untranslated region
