# Supplementary material for: Circulating microRNA’s as a diagnostic tool for hepatocellular carcinoma in a hyper endemic HIV setting, KwaZulu-Natal, South Africa: a case control study protocol focusing on viral etiology
Source: BMC Cancer. 2017 Dec 28;17:894. doi: 10.1186/s12885-017-3915-z (PMC5745691; doi:10.1186/s12885-017-3915-z)
Supplement: Additional file 1: Table S1. — MiRNA deregulation, targets and effect in HCC. (DOCX 12 kb) [file 12885_2017_3915_MOESM1_ESM.docx]

**Table S1:** MiRNA deregulation, targets and effect in HCC

| **HCC Marker** | **Change** | **Comment and speculation** | **Reference** |
| --- | --- | --- | --- |
| MiR-10a/b  MiR-21  MiR-23a/b  MiR-25  MiR-26a  MiR-27a  MiR-122  MiR-125b  MiR-150  MiR-192  MiR-222  MiR-223  MiR-342-3p  MiR-375  MiR-423  MiR-500  MiR-718  MiR-885-5p  MiR-Let-7f | up  up  down  up  down  up  down  down  down  up  up  down  up  down  up  up  down  up  down | Targets *mib1* to regulate angiogenesis  Regulates *PTEN* tumour suppressor gene  Inhibition of topoisomerase 1 expression  Inhibiting *RhoGDI1* to promote proliferation  Targets IL-6-Stat3 signalling/activates tumour suppressor  Targets *PLK2*, apoptosis, promotes proliferation  Targets inhibition of Cyclin G1  *Bcl-W, Mcl-1, IL-6R*, regulator of apoptosis  Regulator of cyclin D1, regulates c-Myb protein  Targets *DHFR* and affects p53 tumour suppressor system  Down regulating p27 promotes proliferation  Expression of Stathmin1 reduced, tumour suppressor Regulation NF-κB pathway to promote cell proliferation. Targets AEG-1, promotes proliferation, invasion  Targets p21Cip1/Waf1 and G(1)/S, promotes cell growth  NF_K_A/B activation, repressed CYLD, OTUD7B, TAXIBPI increased tumour aggressiveness, *HOXB8 target gene*  Targets CDK2 and MCM5, targets VCAMI  Regulator of c-myc, apoptosis, proliferation | Wang et al, 2016  Meng et al, 2007  Wang et al, 2013  Wang et al, 2015  Yang et al, 2013  Tian et al, 2014  Gramantieri et al, 2007  B-Strathmann, 2014  Zhang et al, 2012  Song et al, 2008  Yang et al, 2014  Wong et al, 2008  Zhao and Zhang, 2015  He et al, 2012  Lin et al, 2011  Zhang et al, 2015  Sugimachi et al 2014  Afanasyeva, 2011  Sun et al, 2013 |
